# 2-Methyl-6-(trifluoro­meth­yl)imidazo[1,2-*a*]pyridine-3-carbonitrile

**DOI:** 10.1107/S1600536811003928

**Published:** 2011-02-05

**Authors:** Hoong-Kun Fun, Mohd Mustaqim Rosli, D. J. Madhu Kumar, D. Jagadeesh Prasad, G. K. Nagaraja

**Affiliations:** a X-ray Crystallography Unit, School of Physics, Universiti Sains Malaysia, 11800 USM, Penang, Malaysia; b Department of Chemistry, Mangalore University, Mangalore, Karnataka, India

## Abstract

In the title compound, C_10_H_6_F_3_N_3_, the imidazo[1,2-*a*]pyridine group is essentially planar with a maximum deviation of 0.021 (1) Å. The F atoms in the trifluoro­methyl group and the methyl H atoms are each disordered over two sets of sites with refined site occupancies of 0.68 (1):0.32 (1). In the crystal, mol­ecules are linked into infinite chains through two C—H⋯N inter­actions forming *R*_2_^2^(12) and *R*_2_^2^(8) hydrogen-bond ring motifs. These chains are stacked along the *a* axis.

## Related literature

For the biological activity of imidazole derivatives, see: Biftu *et al.* (2006 ▶); Elhakmoui *et al.* (1994 ▶); Fisher & Lusi (1972 ▶); Gudmundsson & Johns (2003 ▶, 2007 ▶); Kaminski *et al.* (1989 ▶); Rewankar *et al.* (1975 ▶); Rupert *et al.* (2003 ▶). For graph-set descriptions of hydrogen-bond ring motifs, see: Bernstein *et al.* (1995 ▶).
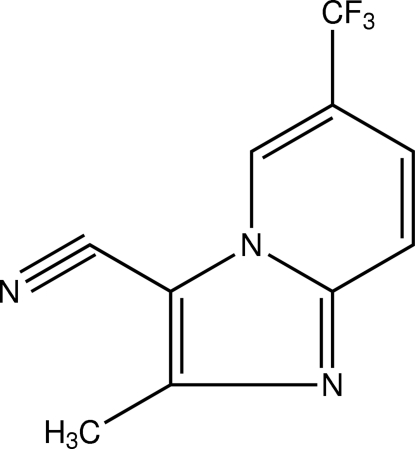


## Experimental

### 

#### Crystal data

C_10_H_6_F_3_N_3_
*M* *_r_* = 225.18
Monoclinic, 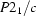

*a* = 5.6871 (3) Å
*b* = 8.5437 (5) Å
*c* = 20.5403 (13) Å
β = 96.653 (4)°
*V* = 991.31 (10) Å^3^
*Z* = 4
Mo *K*α radiation
μ = 0.13 mm^−1^
*T* = 297 K
0.43 × 0.22 × 0.07 mm

#### Data collection

Bruker APEXII DUO CCD area-detector diffractometer
Absorption correction: multi-scan (*SADABS*; Bruker, 2009 ▶) *T*_min_ = 0.945, *T*_max_ = 0.991
10175 measured reflections
2820 independent reflections
1720 reflections with *I* > 2σ(*I*)
*R*_int_ = 0.029

#### Refinement

*R*[*F* ^2^ > 2σ(*F* ^2^)] = 0.049
*wR*(*F* ^2^) = 0.130
*S* = 1.03
2820 reflections
176 parameters
H-atom parameters constrained
Δρ_max_ = 0.13 e Å^−3^
Δρ_min_ = −0.22 e Å^−3^

### 

Data collection: *APEX2* (Bruker, 2009 ▶); cell refinement: *SAINT* (Bruker, 2009 ▶); data reduction: *SAINT*; program(s) used to solve structure: *SHELXTL* (Sheldrick, 2008 ▶); program(s) used to refine structure: *SHELXTL*; molecular graphics: *SHELXTL*; software used to prepare material for publication: *SHELXTL* and *PLATON* (Spek, 2009 ▶).

## Supplementary Material

Crystal structure: contains datablocks global, I. DOI: 10.1107/S1600536811003928/fl2329sup1.cif

Structure factors: contains datablocks I. DOI: 10.1107/S1600536811003928/fl2329Isup2.hkl

Additional supplementary materials:  crystallographic information; 3D view; checkCIF report

## Figures and Tables

**Table 1:** Hydrogen-bond geometry (Å, °)

*D*—H⋯*A*	*D*—H	H⋯*A*	*D*⋯*A*	*D*—H⋯*A*
C1—H1*A*⋯N3^i^	0.93	2.45	3.384 (2)	176
C4—H4*A*⋯N2^ii^	0.93	2.53	3.428 (2)	163

Symmetry codes: (i) 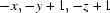; (ii) 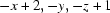.

**Fig. 1.**
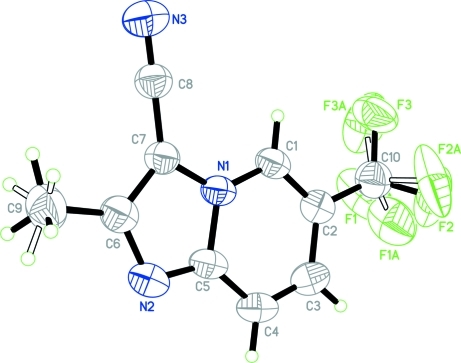
The molecular structure of (I), showing 30% probability displacement ellipsoids and the atom-numbering scheme. Open bonds show minor components.

**Fig. 2.**
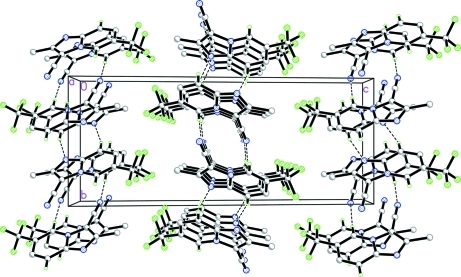
The crystal packing of (I) viewed along the a axis showing molecular chains stacked down the a-axis. Dashed lines indicate hydrogen bonds. H atoms not involved in the hydrogen bond interactions have been omitted for clarity. Only major components are shown.

## supplementary crystallographic information

### Comment

The imidazole nucleus is a widely used pharmacophore in medicinal compounds due to its broad spectrum of biological activities. Moreover imidazole derivatives are isosteres of naturally-occurring nucleotides which allow them to interact easily with the biopolymers of the living systems. It has been known that imidazo[1,2-a]pyridine derivatives exhibit diverse biological activities (Gudmundsson & Johns, 2003) and were used as antiviral (Elhakmoui *et al.*, 1994), antiulcer (Kaminski *et al.*, 1989), antibacterial (Rewankar *et al.*, 1975), antifungal (Fisher & Lusi, 1972), antiprotozoal (Biftu *et al.*, 2006), antiherpes (Gudmundsson & Johns, 2007) and anti-inflammatory (Rupert *et al.*, 2003) compounds.

All bond lengths and angles in the compound are within normal range. The imidazo[1,2-a] pyridine group is planar with maximum deviation of -0.021 (1)Å for atom N1 (Fig. 1). The F atoms in the trifluoromethyl group and the methyl H atoms are disordered over two positions with refined site occupancies of 0.68 (1):0.32 (1).

In the crystal structure, the molecules form infinite chains through C1—H1A···N3^i^ and C4—H4A···N2^ii^ (Table 1) interactions. These interactions also form R^2^_2_(12) and R^2^_2_(8) hydrogen ring motifs, respectively (Bernstein *et al.*, 1995). The chains are stacked along the *a*-axis (Fig. 2).

### Experimental

A mixture of 5-(trifluoromethyl) pyridin-2-amine (0.01mol) and dimethylacetamide dimethyl acetal (0.03mol) was refluxed for 24 hr at 900 C. The resultant product was recrystallized from ethanol. The product so obtained (0.01mol) was refluxed with bromoacetonitrile (0.01mol) in toluene at 600 C. The product was then removed by evaporation of toluene under reduced pressure and it was isolated by column chromatography using ethyl acetate as an eluent. It was then recrystallized by slow evaporation from ethanol to give crystals suitable for x-ray analysis.

### Refinement

H atoms were placed in calculated positions [C–H = 0.93–0.96 Å] and refined as riding with U_iso_(H) = 1.2_eq_(C) or 1.5U_eq_(methyl C). A rotating group model was used for the methyl group. The F atoms in trifluoromethyl group and the H atoms in methyl group are disordered over two position with refined site occupancies of 0.68 (1):0.32 (1).

### Crystal data

**Table:**

C_10_H_6_F_3_N_3_	*F*(000) = 456
*M**_r_* = 225.18	*D*_x_ = 1.509 Mg m^−^^3^
Monoclinic, *P*2_1_/*c*	Mo *K*α radiation, λ = 0.71073 Å
Hall symbol: -P 2ybc	Cell parameters from 2837 reflections
*a* = 5.6871 (3) Å	θ = 2.6–25.3°
*b* = 8.5437 (5) Å	µ = 0.13 mm^−^^1^
*c* = 20.5403 (13) Å	*T* = 297 K
β = 96.653 (4)°	Plate, colourless
*V* = 991.31 (10) Å^3^	0.43 × 0.22 × 0.07 mm
*Z* = 4	

### Data collection

**Table:**

Bruker APEXII DUO CCD area-detector diffractometer	2820 independent reflections
Radiation source: fine-focus sealed tube	1720 reflections with *I* > 2σ(*I*)
graphite	*R*_int_ = 0.029
φ and ω scans	θ_max_ = 29.8°, θ_min_ = 2.6°
Absorption correction: multi-scan (*SADABS*; Bruker, 2009)	*h* = −7→7
*T*_min_ = 0.945, *T*_max_ = 0.991	*k* = −11→11
10175 measured reflections	*l* = −28→28

### Refinement

**Table:**

Refinement on *F*^2^	Primary atom site location: structure-invariant direct methods
Least-squares matrix: full	Secondary atom site location: difference Fourier map
*R*[*F*^2^ > 2σ(*F*^2^)] = 0.049	Hydrogen site location: inferred from neighbouring sites
*wR*(*F*^2^) = 0.130	H-atom parameters constrained
*S* = 1.03	*w* = 1/[σ^2^(*F*_o_^2^) + (0.0575*P*)^2^ + 0.0655*P*] where *P* = (*F*_o_^2^ + 2*F*_c_^2^)/3
2820 reflections	(Δ/σ)_max_ < 0.001
176 parameters	Δρ_max_ = 0.13 e Å^−^^3^
0 restraints	Δρ_min_ = −0.22 e Å^−^^3^

### Special details

**Table:**

Geometry. All esds (except the esd in the dihedral angle between two l.s. planes) are estimated using the full covariance matrix. The cell esds are taken into account individually in the estimation of esds in distances, angles and torsion angles; correlations between esds in cell parameters are only used when they are defined by crystal symmetry. An approximate (isotropic) treatment of cell esds is used for estimating esds involving l.s. planes.
Refinement. Refinement of F^2^ against ALL reflections. The weighted *R*-factor *wR* and goodness of fit S are based on F^2^, conventional *R*-factors *R* are based on F, with F set to zero for negative F^2^. The threshold expression of F^2^ > 2sigma(F^2^) is used only for calculating *R*-factors(gt) *etc*. and is not relevant to the choice of reflections for refinement. *R*-factors based on F^2^ are statistically about twice as large as those based on F, and R– factors based on ALL data will be even larger.

### Fractional atomic coordinates and isotropic or equivalent isotropic displacement parameters (Å^2^)

**Table:**

	*x*	*y*	*z*	*U*_iso_*/*U*_eq_	Occ. (<1)
C10	0.2247 (4)	0.2282 (2)	0.30231 (9)	0.0633 (5)	
F1	0.1174 (15)	0.0950 (4)	0.2794 (3)	0.1138 (18)	0.675 (13)
F2	0.3304 (7)	0.2905 (11)	0.2575 (3)	0.134 (2)	0.675 (13)
F3	0.0427 (9)	0.3205 (5)	0.3079 (2)	0.0858 (13)	0.675 (13)
F1A	0.246 (3)	0.1157 (11)	0.2609 (4)	0.097 (3)	0.325 (13)
F2A	0.329 (2)	0.3390 (12)	0.2678 (6)	0.131 (4)	0.325 (13)
F3A	0.0178 (17)	0.271 (2)	0.3077 (6)	0.141 (5)	0.325 (13)
N1	0.46963 (19)	0.24647 (12)	0.47742 (6)	0.0410 (3)	
N2	0.7873 (2)	0.15187 (14)	0.54049 (6)	0.0528 (3)	
N3	0.1553 (2)	0.49667 (17)	0.57317 (7)	0.0660 (4)	
C1	0.3230 (3)	0.27046 (16)	0.42094 (8)	0.0447 (4)	
H1A	0.1888	0.3328	0.4206	0.054*	
C2	0.3776 (3)	0.20129 (17)	0.36531 (7)	0.0478 (4)	
C3	0.5809 (3)	0.10468 (18)	0.36599 (8)	0.0541 (4)	
H3A	0.6161	0.0581	0.3274	0.065*	
C4	0.7237 (3)	0.07997 (17)	0.42235 (8)	0.0530 (4)	
H4A	0.8554	0.0153	0.4228	0.064*	
C5	0.6712 (2)	0.15294 (16)	0.48021 (8)	0.0455 (4)	
C6	0.6634 (3)	0.24616 (17)	0.57735 (8)	0.0486 (4)	
C7	0.4662 (2)	0.30593 (16)	0.54019 (7)	0.0432 (3)	
C8	0.2918 (3)	0.40991 (17)	0.55779 (7)	0.0468 (4)	
C9	0.7402 (3)	0.2770 (2)	0.64782 (8)	0.0654 (5)	
H9A	0.7533	0.1797	0.6713	0.098*	0.675 (13)
H9B	0.6256	0.3425	0.6654	0.098*	0.675 (13)
H9C	0.8910	0.3287	0.6523	0.098*	0.675 (13)
H9D	0.8904	0.2278	0.6603	0.098*	0.325 (13)
H9E	0.6250	0.2353	0.6738	0.098*	0.325 (13)
H9F	0.7547	0.3878	0.6549	0.098*	0.325 (13)

### Atomic displacement parameters (Å^2^)

**Table:**

	*U*^11^	*U*^22^	*U*^33^	*U*^12^	*U*^13^	*U*^23^
C10	0.0701 (12)	0.0666 (11)	0.0522 (10)	0.0091 (10)	0.0024 (9)	−0.0014 (8)
F1	0.131 (3)	0.0749 (15)	0.117 (3)	0.001 (2)	−0.065 (3)	−0.0194 (17)
F2	0.085 (2)	0.255 (7)	0.0643 (18)	0.003 (3)	0.0218 (15)	0.049 (3)
F3	0.093 (3)	0.095 (2)	0.0627 (18)	0.0467 (16)	−0.0183 (18)	−0.0041 (12)
F1A	0.116 (6)	0.093 (4)	0.071 (3)	0.011 (4)	−0.032 (4)	−0.018 (3)
F2A	0.213 (10)	0.090 (5)	0.082 (5)	−0.027 (4)	−0.018 (5)	0.050 (3)
F3A	0.039 (3)	0.302 (15)	0.081 (5)	0.003 (6)	0.002 (3)	0.042 (8)
N1	0.0356 (6)	0.0390 (6)	0.0485 (7)	0.0048 (5)	0.0053 (5)	0.0016 (5)
N2	0.0445 (7)	0.0524 (7)	0.0604 (8)	0.0094 (6)	0.0009 (6)	0.0029 (6)
N3	0.0590 (8)	0.0662 (9)	0.0748 (10)	0.0127 (7)	0.0158 (7)	−0.0053 (7)
C1	0.0389 (7)	0.0421 (7)	0.0524 (9)	0.0057 (6)	0.0024 (7)	0.0051 (6)
C2	0.0468 (8)	0.0463 (8)	0.0498 (9)	0.0012 (6)	0.0043 (7)	0.0004 (6)
C3	0.0525 (9)	0.0530 (9)	0.0580 (10)	0.0038 (7)	0.0117 (8)	−0.0084 (7)
C4	0.0432 (8)	0.0486 (8)	0.0680 (10)	0.0103 (7)	0.0096 (8)	−0.0042 (7)
C5	0.0371 (7)	0.0400 (7)	0.0593 (9)	0.0067 (6)	0.0046 (7)	0.0022 (6)
C6	0.0451 (8)	0.0471 (8)	0.0532 (9)	0.0008 (6)	0.0045 (7)	0.0036 (6)
C7	0.0403 (7)	0.0434 (7)	0.0462 (8)	0.0040 (6)	0.0064 (6)	0.0027 (6)
C8	0.0435 (8)	0.0466 (8)	0.0509 (8)	0.0023 (6)	0.0078 (7)	0.0010 (6)
C9	0.0651 (11)	0.0746 (12)	0.0540 (10)	0.0025 (9)	−0.0037 (9)	0.0045 (8)

### Geometric parameters (Å, °)

**Table:**

C10—F3A	1.249 (10)	C2—C3	1.420 (2)
C10—F2	1.273 (4)	C3—C4	1.351 (2)
C10—F1A	1.298 (7)	C3—H3A	0.9300
C10—F3	1.316 (4)	C4—C5	1.405 (2)
C10—F1	1.350 (5)	C4—H4A	0.9300
C10—F2A	1.359 (9)	C6—C7	1.379 (2)
C10—C2	1.491 (2)	C6—C9	1.486 (2)
N1—C1	1.3635 (19)	C7—C8	1.4095 (19)
N1—C7	1.3880 (18)	C9—H9A	0.9600
N1—C5	1.3931 (17)	C9—H9B	0.9600
N2—C5	1.3340 (19)	C9—H9C	0.9600
N2—C6	1.3571 (19)	C9—H9D	0.9600
N3—C8	1.1442 (17)	C9—H9E	0.9600
C1—C2	1.354 (2)	C9—H9F	0.9600
C1—H1A	0.9300		
			
F3A—C10—F1A	115.7 (7)	C2—C3—H3A	119.8
F2—C10—F3	104.8 (4)	C3—C4—C5	119.33 (13)
F2—C10—F1	109.4 (4)	C3—C4—H4A	120.3
F3—C10—F1	102.0 (4)	C5—C4—H4A	120.3
F3A—C10—F2A	108.3 (9)	N2—C5—N1	111.00 (12)
F1A—C10—F2A	95.5 (7)	N2—C5—C4	130.66 (13)
F3A—C10—C2	115.4 (6)	N1—C5—C4	118.32 (14)
F2—C10—C2	114.5 (3)	N2—C6—C7	110.63 (14)
F1A—C10—C2	111.4 (4)	N2—C6—C9	122.40 (14)
F3—C10—C2	113.7 (3)	C7—C6—C9	126.97 (14)
F1—C10—C2	111.5 (2)	C6—C7—N1	106.29 (12)
F2A—C10—C2	108.4 (5)	C6—C7—C8	129.98 (14)
C1—N1—C7	131.48 (12)	N1—C7—C8	123.71 (13)
C1—N1—C5	122.71 (12)	N3—C8—C7	178.02 (17)
C7—N1—C5	105.77 (12)	C6—C9—H9A	109.5
C5—N2—C6	106.31 (12)	C6—C9—H9B	109.5
C2—C1—N1	118.37 (13)	C6—C9—H9C	109.5
C2—C1—H1A	120.8	C6—C9—H9D	109.5
N1—C1—H1A	120.8	C6—C9—H9E	109.5
C1—C2—C3	120.76 (15)	H9D—C9—H9E	109.5
C1—C2—C10	119.84 (14)	C6—C9—H9F	109.5
C3—C2—C10	119.40 (14)	H9D—C9—H9F	109.5
C4—C3—C2	120.49 (14)	H9E—C9—H9F	109.5
C4—C3—H3A	119.8		
			
C7—N1—C1—C2	176.78 (13)	C6—N2—C5—N1	−0.64 (16)
C5—N1—C1—C2	−0.6 (2)	C6—N2—C5—C4	177.88 (15)
N1—C1—C2—C3	0.7 (2)	C1—N1—C5—N2	178.33 (12)
N1—C1—C2—C10	−178.43 (14)	C7—N1—C5—N2	0.40 (16)
F3A—C10—C2—C1	−22.2 (11)	C1—N1—C5—C4	−0.4 (2)
F2—C10—C2—C1	120.0 (5)	C7—N1—C5—C4	−178.32 (13)
F1A—C10—C2—C1	−156.7 (8)	C3—C4—C5—N2	−177.24 (15)
F3—C10—C2—C1	−0.4 (4)	C3—C4—C5—N1	1.2 (2)
F1—C10—C2—C1	−115.0 (5)	C5—N2—C6—C7	0.64 (17)
F2A—C10—C2—C1	99.5 (6)	C5—N2—C6—C9	−178.97 (14)
F3A—C10—C2—C3	158.6 (11)	N2—C6—C7—N1	−0.40 (16)
F2—C10—C2—C3	−59.1 (5)	C9—C6—C7—N1	179.19 (14)
F1A—C10—C2—C3	24.1 (8)	N2—C6—C7—C8	−178.74 (14)
F3—C10—C2—C3	−179.6 (3)	C9—C6—C7—C8	0.8 (3)
F1—C10—C2—C3	65.8 (5)	C1—N1—C7—C6	−177.68 (14)
F2A—C10—C2—C3	−79.7 (6)	C5—N1—C7—C6	0.00 (15)
C1—C2—C3—C4	0.1 (2)	C1—N1—C7—C8	0.8 (2)
C10—C2—C3—C4	179.24 (15)	C5—N1—C7—C8	178.48 (13)
C2—C3—C4—C5	−1.0 (2)		

### Hydrogen-bond geometry (Å, °)

**Table:**

*D*—H···*A*	*D*—H	H···*A*	*D*···*A*	*D*—H···*A*
C1—H1A···N3^i^	0.93	2.45	3.384 (2)	176
C4—H4A···N2^ii^	0.93	2.53	3.428 (2)	163

Symmetry codes: (i) −*x*, −*y*+1, −*z*+1; (ii) −*x*+2, −*y*, −*z*+1.
